# Utilization of Mobile Health and Its Associated Factors in Ethiopia: A Systematic Review

**DOI:** 10.1155/bmri/4034726

**Published:** 2026-06-12

**Authors:** Andualem Fentahun Senishaw, Abraham Keffale Mengistu, Bayou Tilahun Assaye, Sefefe Birhanu Tizie, Muluken Belachew Mengistie, Ayenew Sisay Gebeyew, Aynadis Worku Shimie, Maru Meseret Tadele, Tesfaye Shumet Mekonnen, Gizaw Hailiye Teferi

**Affiliations:** ^1^ Department of Health Informatics, College of Medicine and Health Sciences, Debre Markos University, Debre Markos, Ethiopia, dmu.edu.et; ^2^ Department of Public Health, College of Medicine and Health Sciences, Debre Markos University, Debre Markos, Ethiopia, dmu.edu.et

**Keywords:** Ethiopia, mobile health, SMS, systematic review, use of mHealth

## Abstract

**Background:**

Mobile health consists of SMS reminders, smartphone applications, mobile telemedicine, and mobile applications that can improve access to healthcare, improve adherence to the treatment process, and facilitate communication between patients and providers. In Ethiopia, mobile health adoption is growing rapidly; however, there is limited systematic review research on how mobile health is currently being utilized and the factors influencing its use. This systematic review summarizes available studies to evaluate the current level of mobile health utilization in Ethiopia and what factors are related to its utilization.

**Methods:**

A systematic review was performed on PubMed, the Scopus database, African Journals Online, and Google Scholar as of November 25, 2025, for research articles that report the use of mobile health by patients, healthcare professionals, and university students in Ethiopia. The articles were reviewed and evaluated for quality of evidence, and data extraction was done using a standard template and the Joanna Briggs Institute method of determining the quality of evidence.

**Results:**

Utilization rates for mobile health ranged from a low of 13% to a high of 71.2%, with increased levels of use noted for physicians, antiretroviral therapy patients, and students. On the other hand, younger‐aged individuals, those living in urban areas, those who have received a college education, those who have a favorable attitude toward mobile health, and those who have technical skills, perceived usefulness, ease of use, internet access, and availability of technical support were all found to contribute positively to mobile health utilization.

**Conclusion:**

This systematic review identified varying levels of mobile health utilization across different settings and population groups in Ethiopia. Commonly reported factors associated with mHealth use included digital literacy, technical skills, internet access, positive attitudes toward mHealth, and availability of technical support. To increase the level of mobile health use, it is important to improve users′ attitudes, provide technical support, enhance digital skills, and develop necessary infrastructures.

## 1. Introduction

Poor healthcare systems often lead to increased morbidity and mortality, which undermine economic growth and reduce societal productivity [1, 2]. On the other hand, eHealth includes several digital platforms, such as electronic health records, telemedicine, and mobile health (mHealth); in their entirety, these electronic health interventions have the potential to improve quality and increase access to healthcare and health services, ultimately reducing the burden of disease through improved health outcomes [1, 3, 4]. Among these, mHealth services, such as smartphone apps, short message service **(**SMS)‐based reminders, and mobile‐based health information sharing, represent a key component of electronic health, specifically supporting patient engagement, adherence, and real‐time health communication [1, 5, 6]. mHealth is defined as the deployment of portable wireless technologies such as mobile phones and smart devices (including SMS text messaging), applications for mobile devices, and other portable digital technologies [3].

Globally, mHealth has been known as a cost‐effective and scalable digital technology that can improve access to health care services [7]. mHealth programs have proven to improve communications between providers and patients and provide support for maternal and child health services, as well as improve management of chronic diseases and motivate individuals to seek healthcare by providing timely reminders and educational messages [4, 5].

Recent expansion of mobile network coverage and increased mobile phone ownership in sub‐Saharan Africa have made it an optimal environment for integrating mobile technology into existing health care delivery systems [8]. Studies in this region have shown that mobile phone‐based interventions increase attendance at antenatal clinics, adherence to immunization schedules, access to health information, and continuity of care for patients living with HIV/AIDS and other chronic diseases [6, 9, 10].

In Ethiopia, several health system problems, such as poor infrastructure, a limited number of health professionals, and geographic and socioeconomic challenges to access health services, indicate the potential value of mHealth [11]. mHealth was the most popular area of digital health study in Ethiopia. Researchers have studied mHealth in maternal and child health and disease‐related areas such as HIV/AIDS, tuberculosis, noncommunicable diseases, and general health communication/reporting systems [11–13].

Ethiopia has witnessed a rise in mHealth adoption among patients, pregnant women, students, and healthcare providers. They began utilizing mHealth to promote compliance with medications, educate themselves on health issues, monitor diseases, facilitate clinical decision‐making (as well as provide early access to healthcare), and improve their healthcare practices [14–17]. Using mHealth allows healthcare providers, patients, and students to access healthcare information as well as to help support their clinical practice [18, 19]. mHealth offers an effective intervention option for pregnant women by sending them reminders and providing guidance to increase their use of maternal health services [20, 21]. Collectively, mHealth is being used to improve healthcare system efficiency and performance and improve health outcomes for the target populations, including patients, healthcare providers, and students. These groups are considered key stakeholders in mHealth utilization because they directly interact with mobile and digital health interventions in routine care [22, 23]. This offers a possible solution for current challenges to improving access to care in Ethiopia [14, 19].

Although Ethiopia is poised to benefit from the adoption of mHealth, the uptake has been inconsistent and has encountered challenges that include weak infrastructure (lack of electricity and/or network coverage), low levels of digital literacy and poor user training, inadequate institutional support and integration capabilities, and challenges related to establishing and expanding sustainable mHealth programs [15, 16, 20, 21].

A systematic review, which documents the range of studies conducted in Ethiopia in terms of geographical location/setting, study group/population, and health service provision, is needed to provide an in‐depth assessment of mHealth utilization in Ethiopia and its correlates/associative factors. Therefore, this systematic review is aimed at systematically reviewing and synthesizing available evidence on the utilization of mHealth and its associated factors in Ethiopia. The findings will enable the Ministry of Health (MOH) and its agencies, program managers, mHealth implementers, and other stakeholders to identify where mHealth adoption has failed, where it is being utilized, and who is using it; provide guidance to program planners about how and where to roll out mHealth interventions at scale; and inform the public health community about the need for increased investment in mHealth infrastructure, systems, and services for improved health equity and health outcomes.

## 2. Methods and Materials

### 2.1. Search Strategy

This study followed the Preferred Reporting Items for Systematic Reviews and Meta‐Analyses (PRISMA) guidelines in its design and reporting [24]. A PRISMA flow diagram was also utilized to summarize the study selection process, including the identification, inclusion, and exclusion of studies. The systematic review protocol for this review was registered at https://www.crd.york.ac.uk/PROSPERO/view/CRD42022344720.

The use of mHealth to enhance healthcare services in Ethiopia was the subject of an electronic systematic literature search. The following databases were searched: PubMed, Scopus, African Journals Online, and Google Scholar. Snowballing was also used to search for potentially pertinent studies in the references of reputable publications. Keywords, free text search queries, and Medical Subject Headings (MeSH) terms were all used in the search. To find publications in online databases, a comprehensive search based on the following combination of search phrases was used: (use OR “utilization”) AND (“mobile health” OR “m‐health” OR “smartphone” OR “short message service” OR “SMS” OR “cell phone” OR “mobile phone” OR “mobile application”) AND (“health”) AND (“associated factors” OR “determinants”) AND (“Ethiopia”).

### 2.2. Study Selection and Eligibility Criteria

Original studies conducted in Ethiopia that reported on the use of mHealth or related mobile technologies to support health service delivery or health outcomes were included in the systematic review. All cross‐sectional and other observational studies (cohort and case‐control) published up until November 25, 2025, were taken into consideration for inclusion. This study includes free full‐text English‐language articles that were published in peer‐reviewed journals or as freely accessible publications through snowballing and were all included in this analysis. This study included data gathered in Ethiopia and a full report on the use of mHealth. However, studies were excluded if they were not conducted in Ethiopia, were not available in full text, or did not report sufficient data relevant to mHealth utilization. Additionally, nonoriginal articles such as case reports, conference proceedings, expert opinions, editorials, reviews, letters, and commentaries were excluded due to their limited methodological rigor and lack of primary data.

### 2.3. Data Extraction

Two authors (A.F.S. and G.H.T.) independently conducted the data extraction processes using a structured Microsoft Excel template. During the screening phase, titles and abstracts of all retrieved records were independently reviewed by the two authors based on the predefined eligibility criteria. To guarantee uniformity and comprehensiveness among the included studies, data extraction was done carefully, employing a structured format. The authors′ names, the year of publication, the region/zone, the study area, the study design, the study population/subject, the sample size, the study purpose, and factors influencing mHealth utilization were extracted from each article. To ensure accuracy, extracted data were cross‐checked by additional reviewers. Discrepancies were resolved through discussion and consensus. The final dataset was reviewed for completeness and consistency prior to analysis.

### 2.4. Quality Appraisal

Each included study was assessed for methodological quality using a structured approach. Two authors (A.F.S. and B.T.A.) independently appraised each study to ensure consistency and reduce bias. The assessment was conducted using the Joanna Briggs Institute (JBI) quality assessment tool, which applies nine predefined criteria [25]. This tool evaluates several aspects of methodological rigor to ensure the methodological strength and bias risk of each study transparently. For each criterion, studies were rated as “yes” or “not appropriate.” An overall quality score was then calculated by summing the number of “yes” responses. Based on this scoring system, studies that achieved a score of 5 or higher out of 9 were of acceptable methodological quality. Therefore, this study achieved acceptable quality by achieving a score of over five out of nine. A more thorough interpretation of the review′s conclusions was made possible by this quality appraisal procedure, which also enhanced the evidence base′s overall validity.

## 3. Results

### 3.1. Included Studies

**Figure 1:**
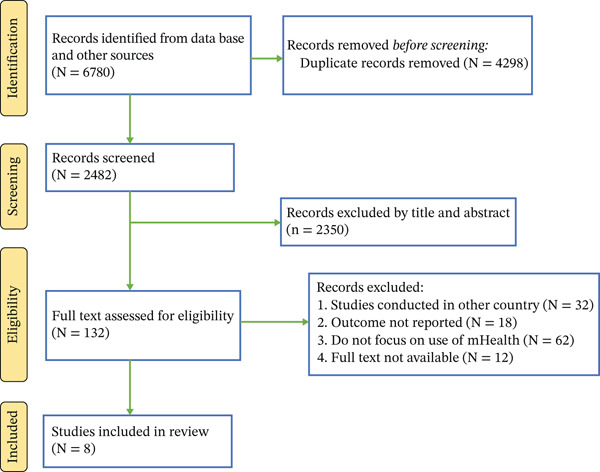
PRISMA flowchart showing the selection process of the articles.

A total of 6780 articles related to the use and determinants of mHealth in Ethiopia were identified across different databases. After removing 4298 duplicates, consequently, we excluded 2350 records based on title and abstract screening. An additional 124 articles were excluded for being studies conducted out of Ethiopia, having an outcome not reported, focusing on the use of mHealth, or being unable to access the full text. The remaining eight full‐text articles were included in the final systematic review (Figure 1).

### 3.2. Characteristics of Included Studies

Among the eight included cross‐sectional studies on mHealth utilization in Ethiopia, the outcome of six studies was predominantly measured as binary variables (yes/no), whereas the remaining two studies employed multiple Likert‐scale items [18, 19], which were then averaged to generate a composite score. These studies were conducted in different regions of Ethiopia, including Addis Ababa [14]; Amhara (Gondar and Debre Markos) [15, 16, 18, 19, 21]; Oromia (Jimma) [20]; and southern regions (Mizan Teferi) [17]. The studies involved a total of 2791 participants, drawn from various population groups such as patients, university students, and health professionals. Sample sizes ranged from 213 in a study conducted on diabetic patients [17] to 423 in a study conducted on hypertension patients [15]. The study participants were recruited using different sampling techniques, including simple random sampling, systematic random sampling, stratified sampling, convenience sampling, and census methods. Across the studies, participants′ engagement with mHealth for receiving health information, getting reminders, or communicating with health providers varied according to population group, setting, and access to technology (Table 1).

**Table 1:** Characteristics of included studies.

Author and year of publication	Setting/area	Study subject	Sample size	Sampling technique	Purpose of study/use
Guracho et al. [14]	Addis Ababa Hospital	Patient	419	Convenient	For mental health
Hailiye Teferi, et al. [18]	Debre Markos University	Student	405	Stratified sampling	For various health purposes
Teferi, et al. [19]	Amhara Region Hospitals	Physician	417	Simple random sampling	For clinical practice
Teferi, et al. [15]	Gondar health facilities	Patient	423	Simple random sampling	For medication reminders
Kebede et al.	Gondar Hospital	Patient	316	Systematic random sampling	For medication reminders
Abdissa et al. [20]	Jimma Primary Health Care Unit	Patient	279	Simple random sampling	To get health information
Endehabtu et al. [21]	Gondar health facilities	Patient	319	Systematic random sampling	For health‐related information
Bogale et al. [17]	Mizan Tipi Hospital	Patient	213	Census	For medication reminders

### 3.3. Magnitude of mHealth Use

This study demonstrated marked variation in the level of mHealth use across different population groups in Ethiopia, with consistent patterns emerging in the magnitude of utilization of mHealth. Among patients and healthcare professionals, particularly antiretroviral therapy (ART) patients [16] and physicians working in hospital settings [19], utilization of mHealth was high when compared with other included studies. In contrast, mHealth use among general hospital patients remained considerably lower, and also, patients′ engagement with health‐related services such as appointment reminders, treatment follow‐up, and health information access through mobile platforms was limited [17, 20]. The lowest level of mHealth use was also observed among individuals with mental health conditions [14].

### 3.4. Factors Associated With the Use of mHealth

Even though most of the studies did not address factors associated with the use of mHealth, some of the studies checked the presence of an association between several factors. Educational level was a factor associated with mHealth use, where higher education increased the likelihood of mHealth utilization [14]. Urban residence also emerged as a significant contextual factor [14]. Age was reported as a determinant, with younger participants more likely to use mHealth services [18].

Attitudes of participants to use mobile applications were reported in two studies, and they are strongly associated with mHealth use [14, 19]. Additionally, technical skill and the presence of institutional technical support were reported as significant factors in the use of mHealth services [18, 19]. Access to the internet or network connectivity, prior computer training, and perceived usefulness of mobile applications were also positively correlated with mHealth use among physicians [19]. The easiness of the mobile application to use and its trustworthiness were also associated factors in using mHealth [18].

## 4. Discussion

In this systematic review, mHealth comprises the use of mobile phones, smartphones, SMS, and mobile applications to support health service delivery, health communication, and appointment reminders. Although a growing number of studies in Ethiopia have examined the utilization of mHealth and factors influencing its usage among different population groups, the available evidence remains scattered and inconsistent across settings and study populations. This has resulted in a limited, fragmented understanding of the mHealth use and enabling factors influencing mHealth use in the Ethiopian health care context. To date, no comprehensive systematic review has specifically gathered and synthesized the existing evidence on the use of mHealth and the commonly reported factors affecting mHealth utilization in Ethiopia.

The synthesis of evidence from the included eight studies suggests that the magnitude of mHealth usage in Ethiopia varied across the studies, ranging from 13% to 71.2%. A possible reason for the discrepancy is due to differences in study populations, settings, and measurement approaches. Evidence suggests that disparities in infrastructure, access to mobile devices, and availability of technical support influence adoption across settings [26]. Higher utilization is often observed among urban, educated, and digitally literate populations, where access to the internet and technological resources is greater [26]. Furthermore, heterogeneity in the definitions and outcome measurements contributes to inconsistencies in reported utilization levels across studies, with some using binary indicators and others applying Likert‐scale‐based composite measures. Studies using broader definitions of utilization are more likely to report higher prevalence, whereas those applying stricter or scaled measurements may report lower levels of utilization. This inconsistency suggests that mHealth implementation is not yet standardized or fully integrated, highlighting the fragmented nature of mHealth use in Ethiopia and its early or transitional stage of development.

Across the included studies, relatively higher levels of mHealth use were reported in studies conducted on physician and ART patients when compared with other included studies [16, 19]. This study is consistent with a study conducted in the United Kingdom [27] and with a scoping review [28, 29]. The higher utilization observed in these studies may be explained by physicians having better access to digital infrastructure, stronger mobile network coverage, and greater exposure to digital health interventions. Similarly, studies conducted on Debre Markos University students reported relatively high levels of mHealth utilization for various health purposes. In addition, other studies conducted in Ghana support this result [30].

Studies conducted in hypertension patients and pregnant mothers reported moderate levels of mHealth use. On the other hand, lower levels of mHealth utilization were consistently reported in studies conducted in more peripheral areas. For instance, a study conducted in Mizan Tepi [17] found limited use of mHealth among patients despite the growing national emphasis on digital solutions. Likewise, a study conducted in Jimma′s primary healthcare units [20] documented relatively low uptake of mobile‐based health services by clients. Similar low uptake has been reported in multiple low‐ and middle‐income countries [31, 32]. A possible explanation for lower utilization of mHealth may be due to the study setting being semiurban, which can limit the access to mobile and digital technology initiatives as well as network access. In addition, a lower level of mHealth utilization is reported in studies conducted in Addis Ababa [14]. This study is consistent with another pooled study result conducted on mental health patients [33].

Across the reviewed studies, age consistently emerged as an important determinant of mHealth utilization. Younger participants, particularly those with greater exposure to digital technologies, were more likely to engage with mHealth. This finding is consistent with the literature at large, where younger age cohorts appear to be generally more accepting of new technological advances and more skilled with digital media [34]. Urban residents were reported as associated with mHealth use. A possible explanation may be that urban residents can easily get in touch with the latest available technology related to mHealth, and they are more aware of mHealth and digital technology than rural residents [35].

Educational status was also identified as a commonly reported factor influencing mHealth use. Participants with higher levels of education were consistently reported to be more likely to utilize mHealth services compared with those with limited formal education; this is also explained in another systematic review finding [26]. This association may be explained by increased health literacy, improved confidence in interpreting digital health information, and better capacity to navigate mobile applications among individuals with higher educational attainment.

Perceived usefulness and ease of use were also considered as behavioral predictors of mHealth utilization, and attitude plays a critical role in the sustained use of mHealth across health settings. Studies from multiple regions report that the majority of healthcare workers perceive mHealth as useful, efficient, and supportive for clinical work, particularly in countries with established digital systems [36]. Participants who believed that mHealth could improve health communication, facilitate appointment reminders, enhance access to information, or support treatment adherence were more likely to use such services.

Another dominant factor across the reviewed studies was the strong association between technical skill and mHealth use. Furthermore, the availability of technical support was associated with higher levels of utilization. Healthcare workers with higher digital literacy are more confident and consistent in using mHealth tools, whereas those with limited skills often struggle, reducing utilization. Likewise, the availability of technical support consistently reported association across studies influences sustained use, as timely assistance prevents frustration from system errors or connectivity issues.

## 5. Limitations

All included studies were cross‐sectional and relied on self‐reported data, making it difficult to establish causal relationships, which limited causal inference. In addition, the results of the included studies varied due to differences in the operationalization and measurement of mHealth use; therefore, caution is needed when interpreting results across different populations and regions.

## 6. Conclusion

This systematic review identified varying levels of mHealth utilization across different settings and population groups in Ethiopia. Commonly reported factors associated with mHealth utilization included technical skills, digital literacy, prior exposure to mobile technologies, positive attitudes toward mHealth, internet access, and the availability of technical support. The findings suggest that improving digital competence, strengthening technical support, and enhancing infrastructure may help improve mHealth utilization in Ethiopia. In addition, efforts such as digital literacy initiatives, health provider training, and institutional support could contribute to better integration of mHealth into healthcare delivery, particularly in underserved settings. By addressing these barriers and strengthening enabling conditions, practice on the utilization of mHealth can be enhanced, which significantly enhances access, continuity, and quality of healthcare services, particularly in remote and underserved communities.

**Nomenclature**
ART: Antiretroviral therapy
AIDS: Acquired Immunodeficiency Syndrome
HIV: Human immunodeficiency virus
JBI: Joanna Briggs Institute
mHealth: Mobile Health
MeSH: Medical Subject Headings
MOH: Ministry of Health
PRISMA: Preferred Reporting Items for Systematic Reviews and Meta‐Analyses
PubMed: Public/Publisher MEDLINE
SMS: Short Message Service

## Author Contributions

A.F.S. made significant contributions to the data extraction, analysis, quality appraisal, and drafting and writing of the manuscript. G.H.T. contributed to data extraction, revising the manuscript, and analysis; B.T.A. and M.M.T. contributed to revising the manuscript, quality appraisal, and analysis. A.K.M., S.B.T., M.B.M., A.S.G., A.W.S., and T.S.M. contributed extensively to revising the manuscript and analysis.

## Funding

No funding was received for this manuscript.

## Disclosure

All authors read and approved the final manuscript.

## Conflicts of Interest

The authors declare no conflicts of interest.

## Data Availability

The data used during the current study are available from the corresponding author upon reasonable request.

## References

[1] Alkhuzaimi F. , Rainey D. , Wilson C. B. , and Bloomfield J. , The Impact of Mobile Health Interventions on Service Users′ Health Outcomes and the Role of Health Professions: A Systematic Review of Systematic Reviews—Protocol, Systematic Reviews. (2024) 13, no. 1, 10.1186/s13643-024-02624-y, 39068478. PMC11283682 39068478

[2] Tegegne M. D. and Wubante S. M. , Identifying Barriers to the Adoption of Information Communication Technology in Ethiopian Healthcare Systems. A Systematic Review, Advances in Medical Education and Practice. (2022) 13, 821–828, 10.2147/AMEP.S374207, 35959138. 35959138 PMC9362847

[3] Sakib A. M. , Alam S. A. , and Islam A. , User-Centered Design and Validation of mHealth App for Providing Vital Assistance and Emergency Healthcare Support in Bangladesh, 2024 International Congress on Human-Computer Interaction, Optimization and Robotic Applications (HORA), 2024, IEEE.

[4] Amoakoh-Coleman M. , Borgstein A. B. , Sondaal S. F. , Grobbee D. E. , Miltenburg A. S. , Verwijs M. , Ansah E. K. , Browne J. L. , and Klipstein-Grobusch K. , Effectiveness of mHealth Interventions Targeting Health Care Workers to Improve Pregnancy Outcomes in Low- and Middle-Income Countries: A Systematic Review, Journal of Medical Internet Research. (2016) 18, no. 8, 10.2196/jmir.5533, 27543152. PMC5010646 27543152

[5] Lee S. H. , Nurmatov U. B. , Nwaru B. I. , Mukherjee M. , Grant L. , and Pagliari C. , Effectiveness of mHealth Interventions for Maternal, Newborn and Child Health in Low- and Middle-Income Countries: Systematic Review and Meta-Analysis, Journal of Global Health. (2016) 6, no. 1, 010401, 10.7189/jogh.06.010401, 26649177. 26649177 PMC4643860

[6] Sondaal S. F. , Browne J. L. , Amoakoh-Coleman M. , Borgstein A. , Miltenburg A. S. , Verwijs M. , and Klipstein-Grobusch K. , Assessing the Effect of mHealth Interventions in Improving Maternal and Neonatal Care in Low- and Middle-Income Countries: A Systematic Review, PLoS One. (2016) 11, no. 5, e0154664, 10.1371/journal.pone.0154664, 27144393. 27144393 PMC4856298

[7] WHO , mHealth: New Horizons for Health Through Mobile Technologies, 2011, World Health Organization.

[8] GSMA , The Mobile Economy Africa 2024, 2024, GSMA.

[9] Kachimanga C. , Zaniku H. R. , Divala T. H. , Ket J. C. , Mukherjee J. S. , Palazuelos D. , Kulinkina A. V. , Abejirinde I. O. O. , and Akker T. , Evaluating the Adoption of mHealth Technologies by Community Health Workers to Improve the Use of Maternal Health Services in Sub-Saharan Africa: Systematic Review, JMIR mHealth and uHealth. (2024) 12, e55819, 10.2196/55819, 39316427. 39316427 PMC11462100

[10] Or C. K. , Liu K. , So M. K. P. , Cheung B. , Yam L. Y. C. , Tiwari A. , Lau Y. F. E. , Lau T. , Hui P. S. G. , Cheng H. C. , Tan J. , and Cheung M. T. , Improving Self-Care in Patients With Coexisting Type 2 Diabetes and Hypertension by Technological Surrogate Nursing: Randomized Controlled Trial, Journal of Medical Internet Research. (2020) 22, no. 3, e16769, 10.2196/16769, 32217498. 32217498 PMC7148548

[11] Tarekegn S. M. , Tadesse D. , Argaw M. D. , Semahegn A. , Taddesse L. , Shifarraw S. , Enbiale W. , Muluneh M. D. , Abate B. , Tamire A. , and Makonnen M. , Capacity and Performance of Primary Health Care in Ethiopia: A Novel Mixed Methods Measurement in Low-Income Country, BMC Primary Care. (2025) 26, no. 1, 10.1186/s12875-025-02988-7, 41023838. PMC12481810 41023838

[12] Tilahun B. , Gashu K. D. , Mekonnen Z. A. , Endehabtu B. F. , and Angaw D. A. , Mapping the Role of Digital Health Technologies in the Case Detection, Management, and Treatment Outcomes of Neglected Tropical Diseases: A Scoping Review, Tropical Medicine and Health. (2021) 49, no. 1, 10.1186/s41182-021-00307-1, 33618757. PMC7898439 33618757

[13] Manyazewal T. , Woldeamanuel Y. , Blumberg H. M. , Fekadu A. , and Marconi V. C. , The Potential Use of Digital Health Technologies in the African Context: a Systematic Review of Evidence From Ethiopia, NPJ Digital Medicine. (2021) 4, no. 1, 10.1038/s41746-021-00487-4, 34404895. PMC8371011 34404895

[14] Guracho Y. D. , Thomas S. J. , and Win K. T. , Mobile Mental Health Application Use, and App Feature Preferences Among Individuals With Mental Disorders in Ethiopia: A Cross-Sectional Survey, International Journal of Medical Informatics. (2024) 192, 105628, 10.1016/j.ijmedinf.2024.105628, 39288667. 39288667

[15] Zewdu E. M. , Demessie A. , Nigatu A. M. , and Baykemagn N. D. , Intention to Use Mobile Text Message Reminders for Medication Adherence Among Hypertensive Patients in North West Ethiopia: A Cross-Sectional Study, BMC Health Services Research. (2024) 24, no. 1, 10.1186/s12913-024-11794-3, 39578837. PMC11583659 39578837

[16] Kebede M. , Zeleke A. , Asemahagn M. , and Fritz F. , Willingness to Receive Text Message Medication Reminders Among Patients on Antiretroviral Treatment in North West Ethiopia: A Cross-Sectional Study, BMC Medical Informatics and Decision Making. (2015) 15, no. 1, 10.1186/s12911-015-0193-z, 26268394. PMC4535252 26268394

[17] Bogale B. , Habte A. , Haile D. , Guteta M. , Mohammed N. , and Gebremichael M. A. , Willingness to Receive mHealth Messages Among Diabetic Patients at Mizan Tepi University Teaching Hospital: Implications for Digital Health, Patient Preference and Adherence. (2022) 16, 1499–1509, 10.2147/PPA.S364604, 35769337. 35769337 PMC9234188

[18] Hailiye Teferi G. , Tadele M. M. , Tizazu G. , Hordofa Z. R. , Shimie A. W. , Assaye B. T. , Senishaw A. F. , and Tizie S. B. , Utilization of Mobile Health Applications and Determinant Factors Among Health Science Students at Debre Markos University, Northwest Ethiopia in 2022, PLoS One. (2023) 18, no. 7, e0275689, 10.1371/journal.pone.0275689, 37440563. 37440563 PMC10343100

[19] Teferi G. H. , Tilahun B. C. , Guadie H. A. , and Amare A. T. , Smartphone Medical app Use and Associated Factors Among Physicians at Referral Hospitals in Amhara Region, North Ethiopia, in 2019: Cross-Sectional Study, JMIR mHealth and uHealth. (2021) 9, no. 3, e19310, 10.2196/19310, 33769303. 33769303 PMC8096376

[20] Abdissa H. G. , Duguma G. B. , Ababulgu F. A. , Lemu Y. K. , Gerbaba M. , Noll J. , Sori D. A. , and Koricha Z. B. , Pregnant Mother’s Intention to Use Mobile Phone-Based Messaging Interventions for Improving Maternal and Newborn Health Practices in Jimma Zone, Ethiopia, BMC Digital Health. (2024) 2, no. 1, 10.1186/s44247-024-00094-9.

[21] Endehabtu B. , Weldeab A. , Were M. , Lester R. , Worku A. , and Tilahun B. , Mobile Phone Access and Willingness Among Mothers to Receive a Text-Based mHealth Intervention to Improve Prenatal Care in Northwest Ethiopia: Cross-Sectional Study, JMIR Pediatrics and Parenting. (2018) 1, no. 2, e9618, 10.2196/pediatrics.9618. PMC6715064 31518334

[22] Xie Z. , Or C. , and Ye X.-C. , Acceptance of Mobile Health Applications by the General Public: The Roles of Technology Acceptance Model Constructs, Health-App-Specific Factors, and Socio-Demographic Moderators, Applied Ergonomics. (2026) 130, 104655, 10.1016/j.apergo.2025.104655, 41056749. 41056749

[23] Cheung D. S. T. , Or C. K. , So M. K. P. , Ho K. , and Tiwari A. , The Use of eHealth Applications in Hong Kong: Results of a Random-Digit Dialing Survey, Journal of Medical Systems. (2019) 43, no. 9, 10.1007/s10916-019-1422-2. 31338682

[24] Liberati A. , Altman D. G. , Tetzlaff J. , Mulrow C. , Gøtzsche P. C. , Ioannidis J. P. A. , Clarke M. , Devereaux P. J. , Kleijnen J. , and Moher D. , The PRISMA Statement for Reporting Systematic Reviews and Meta-Analyses of Studies That Evaluate Health Care Interventions: Explanation and Elaboration, Journal of Clinical Epidemiology. (2009) 62, no. 10, e1–e34, 10.1016/j.jclinepi.2009.06.006, 19631507. 19631507

[25] JBI , Critical Appraisal Tools, 2021, JBI, https://jbi.global/critical-appraisal-tools.

[26] Yang S. , Cha M. J. , van Kessel R. , Warrier G. , Thrul J. , Lee M. , Yoon J. , Kang D. , and Cho J. , Understanding Inequalities in Mobile Health Utilization Across Phases: Systematic Review and Meta-Analysis, Journal of Medical Internet Research. (2025) 27, e71349, 10.2196/71349, 40811740. 40811740 PMC12352709

[27] Payne K. F. B. , Wharrad H. , and Watts K. , Smartphone and Medical Related App Use Among Medical Students and Junior Doctors in the United Kingdom (UK): a Regional Survey, BMC Medical Informatics and Decision Making. (2012) 12, no. 1, 10.1186/1472-6947-12-121, 23110712. PMC3504572 23110712

[28] Lee M. , Bin Mahmood A. B. S. , Lee E. S. , Smith H. E. , and Tudor C. L. , Smartphone and Mobile App Use Among Physicians in Clinical Practice: Scoping Review, JMIR mHealth and uHealth. (2023) 11, e44765, 10.2196/44765, 37000498. 37000498 PMC10131676

[29] Osei E. , Kuupiel D. , Vezi P. N. , and Mashamba-Thompson T. P. , Mapping Evidence of Mobile Health Technologies for Disease Diagnosis and Treatment Support by Health Workers in Sub-Saharan Africa: A Scoping Review, BMC Medical Informatics and Decision Making. (2021) 21, no. 1, 10.1186/s12911-020-01381-x, 33407438. PMC7789784 33407438

[30] Peprah P. , Abalo E. M. , Agyemang-Duah W. , Gyasi R. M. , Reforce O. , Nyonyo J. , Amankwaa G. , Amoako J. , and Kaaratoore P. , Knowledge, Attitude, and Use of mHealth Technology Among Students in Ghana: A University-Based Survey, BMC Medical Informatics and Decision Making. (2019) 19, no. 1, 10.1186/s12911-019-0947-0, 31718642. PMC6852777 31718642

[31] Chib A. , van Velthoven M. H. , and Car J. , mHealth Adoption in Low-Resource Environments: A Review of the Use of Mobile Healthcare in Developing Countries, Journal of Health Communication. (2015) 20, no. 1, 4–34, 10.1080/10810730.2013.864735, 24673171. 24673171

[32] Weichelt B. P. , Burke R. , Kieke B. , Pilz M. , and Shimpi N. , Provider Adoption of mHealth in Rural Patient Care: Web-Based Survey Study, JMIR Human Factors. (2024) 11, e55443, 10.2196/55443, 38913992. 38913992 PMC11231617

[33] Guracho Y. D. , Thomas S. J. , and Win K. T. , Smartphone Application Use Patterns for Mental Health Disorders: A Systematic Literature Review and Meta-Analysis, International Journal of Medical Informatics. (2023) 179, 105217, 10.1016/j.ijmedinf.2023.105217, 37748330. 37748330

[34] Naslund J. A. and Aschbrenner K. A. , Technology Use and Interest in Digital Apps for Mental Health Promotion and Lifestyle Intervention Among Young Adults With Serious Mental Illness, Journal of Affective Disorders Reports. (2021) 6, 100227, 10.1016/j.jadr.2021.100227. 40027518 PMC11870643

[35] Mahmood A. , Mahmood A. , Kedia S. , and Chang C. F. , Rural-Urban Disparities in Mobile Health Application Ownership and Utilization Among Cancer Survivors, Medical Care. (2025) 63, no. 2, 111–116, 10.1097/MLR.0000000000002092, 39791845. 39791845

[36] Gagnon M. P. , Ngangue P. , Payne-Gagnon J. , and Desmartis M. , m-Health Adoption by Healthcare Professionals: A Systematic Review, Journal of the American Medical Informatics Association. (2016) 23, no. 1, 212–220, 10.1093/jamia/ocv052, 26078410. 26078410 PMC7814918

